# To tier or not to tier: the institutionalization of the World Health Organization’s power to determine pandemic emergency in the amended International Health Regulations (2005)

**DOI:** 10.1186/s41256-025-00438-6

**Published:** 2025-08-29

**Authors:** Yi Zhang, Yida Guo

**Affiliations:** 1 Vanke School of Public Health, Tsinghua University, No. 30 Shuangqing Road, Haidian District, Beijing, 100084 China; 2 Law School, Nankai University, No. 38 Tongyan Road, Jinnan District, Tianjin, 300350 China

**Keywords:** World Health Organization, International Health Regulations, Public Health Emergency of International Concern, Pandemic Emergency, Pandemic Agreement, Global Health Governance

## Abstract

The binary nature of a Public Health Emergency of International Concern (PHEIC) alert was brought to attention during COVID-19, with the COVID-19 IHR Emergency Committee and some States Parties advocating for an intermediate or regional tier of warning. However, the recent amendments to the International Health Regulations (2005) yielded an unexpected outcome: no proposed lower tier was added to the binary alert framework; instead, ‘pandemic emergency’ was introduced as a tier of alert within the PHEIC framework. This paper argues that the influence of introducing a ‘pandemic emergency’ tier within the World Health Organization’s alert framework, as outlined in the amendments to the International Health Regulations (2005), has been underestimated. While a proposed intermediate alert received some support, it is unlikely to function effectively in raising awareness or mobilizing resources. In contrast, a ‘pandemic emergency’ alert—previously framed as a descriptive, non-binding concept —has been perceived as a more effective tool for alerting against communicable disease threats. The formalization of a de facto determination of pandemic emergency results in a de jure expansion of the World Health Organization’s emergency powers, demonstrating what is often termed as a ‘ratchet effect’. Moreover, the amended International Health Regulations (2005) grant the World Health Organization enhanced legal competences, notably in a binding way. The adoption of the World Health Organization Pandemic Agreement could further extend the legal implications of the World Health Organization’s power to determine a pandemic emergency.

## Introduction

The International Health Regulations (2005) (IHR) are a nearly universally recognized, legally binding instrument of the World Health Organization (WHO), with the WHO Director-General (DG)’s determination of a Public Health Emergency of International Concern (PHEIC) serving as its ‘cornerstone’[1]. However, the emergency power to determine a PHEIC remains contentious, with debate centering on whether its nature is fundamentally technical or political [2]. On 1 June 2024, after intensive negotiations during the 77th World Health Assembly (WHA), a 21-month amendment process to the IHR was completed. In a press release, the WHO highlighted four critical changes [3], one of which was the introduction of a new ‘pandemic emergency’ tier within the PHEIC alert mechanism. This new tier—while still functioning as a PHEIC—represents a heightened level of alert specifically for communicable disease risks.

Despite its introduction, the ‘pandemic emergency’ tier has not been as well embraced as the previously proposed—but ultimately not adopted—intermediate tier. Critics argue that granting the DG authority to determine a ‘pandemic emergency’, instead of a lower-level alert, amounts to ‘a change that made no changes’, as it does not address core controversies within the PHEIC framework [4].

This paper takes a different view. It argues that an intermediate alert tier is not a feasible solution, whereas the introduction of the ‘pandemic emergency’ tier has been underestimated. In reality, the amendments achieve the legal normalization of the expansion of the WHO’s emergency powers. Although the WHO is mandated by its Constitution to act as the directing and coordinating authority on international health, political entanglements and budgetary constraints have historically shaped its operations, compromising its autonomy and effectiveness [5]. Building upon the amended IHR as a normative foundation—though not a panacea—a rigorous analysis of the WHO’s emergency power to determine emergency alerts could help strengthen its leadership role and enable it to meet the high expectations placed on it.

## Alert framework: design and analysis

The Working Group on Amendments to the International Health Regulations (2005) (WGIHR) was mandated by the 75th WHA in May 2022 to consider proposed targeted amendments to the IHR. Over 300 amendments were initially submitted by States Parties. Meanwhile, an Intergovernmental Negotiating Body (INB) was tasked with drafting and negotiating a WHO convention, agreement, or other international instrument on pandemic prevention, preparedness and response (Pandemic Agreement). Since the alert framework was a shared concern in both processes, an alert continuum was discussed by both the WGIHR and the INB.

### A continuum-based alert framework: a tiered approach

The WGIHR aimed to address the binary limitations of the existing PHEIC mechanism by establishing a continuum-based, tiered alert framework. Joint sessions between the WGIHR and the INB considered a continuum-based framework with tiers labeled ‘Public Health Alert–PHEIC–Pandemic’ [6]. Initial proposals from some States Parties included a traffic-light approach, introducing a lower tier based on severity or region, allowing the WHO to issue warnings before a public health event reaches the PHEIC threshold.

Following a joint plenary session, the WGIHR was designated to lead the work on the alert continuum [7]. In a subsequent draft released by the WGIHR Bureau [8], the initially proposed ‘intermediate alert’ was replaced with an ‘early action alert’. Pursuant to Article 1 and Article 12 (4ter) of the proposed Bureau text, an ‘early action alert’ means ‘information and non-binding advice’ issued by the DG for events that do not meet the PHEIC threshold. At the upper end of the continuum, a ‘pandemic emergency’ alert refers to a PHEIC that is infectious in nature. When determining a PHEIC, the DG shall also assess whether it constitutes a ‘pandemic emergency’. In fact, a definition of ‘pandemic’ appeared in Article 1, but the term ‘pandemic emergency’ was preferred throughout the Bureau’s text.

In the final text submitted to the 77th WHA, ‘enhanced action advice’ was retained only in Article 12 as non-binding advice, while ‘pandemic emergency’ was maintained with minor revisions [9]. Ultimately, the amended IHR included no intermediate alert. Instead, a higher tier—‘pandemic emergency’—was formally adopted. As such, the PHEIC alert framework was tiered as ‘PHEIC—Pandemic Emergency’, with the latter applying only to communicable PHEICs. The final outcome–excluding intermediate alerts while incorporating an elevated response tier–demonstrates a deliberate effort to calibrate the framework to better address the most consequential public health emergencies.

### A tier of intermediate alert: its drawbacks

The concept of an intermediate alert—a tier intended to engage the international community before an event escalates to a PHEIC—has been debated for nearly a decade, but was ultimately dismissed as ineffective in addressing global health governance gaps. First emerging in recommendations from the 2015 Ebola Interim Assessment Panel, the concept gained attention, receiving further endorsement by the Review Committee on Role of the IHR in the Ebola Outbreak and Response (the 2016 Review Committee for Ebola) [10]. However, it was omitted from subsequent global strategic plans [11]. The COVID-19 pandemic exposed its limitations rather than validating its necessity.

Critics, including the COVID-19 IHR Emergency Committee [12] and the DG [13], argued that the binary nature of the PHEIC was too restrictive, overly simplistic, and not fit for purpose, as exemplified by the WHO’s initial deliberations in January 2020, when COVID-19 was not deemed a PHEIC but quickly escalated to a full-blown emergency within a week. This reignited debate over the need for an intermediate alert for events below the PHEIC threshold. Opinions diverged within the WHO and the academic community [14]. While some proposed a ‘traffic-light’ system, experts noted significant limitations inherent in a multi-tier system for public health emergencies. The 2021 Review Committee on the Functioning of the IHR during the COVID-19 Response (the 2021 Review Committee for COVID-19) extensively evaluated the concept, concluding that a formal intermediate alert would not solve the core issue of inaction due to limited practical advantages [15].

Indeed, a formal intermediate alert is not the solution—early awareness is. In lieu of a formal intermediate tier, the WHO has implemented early warning mechanisms through both normative and operational adaptations. The WHO could also develop informal guidelines under Article 11 of the IHR, which allows it to share information with States Parties, alerting them to potential emergencies in a graduated manner [16]. Furthermore, as noted by the 2023 Review Committee regarding Amendments to the IHR (the 2023 Review Committee for Amendments), the WHO already provides ongoing and updated advice on public health risks and events through the Event Information Site, which effectively fulfills the function of an intermediate alert [17]. WHO’s Disease Outbreak News and the WHO-led Global Outbreak Alert and Response Network also provide information on confirmed acute public health events or potential events of concern. Crucially, they do so without introducing bureaucratic inertia or undermining the urgency of PHEIC determinations.

The central challenge remains the international community’s commitment to mobilizing resources preemptively, regardless of alert tiers. An intermediate alert does not necessarily enhance resource mobilization. For example, the determination of Mpox as a PHEIC did not result in equitable global access to vaccines, let alone improved resource allocation via an intermediate alert [18]. Similarly, funding shortfalls prior to the PHEIC declaration during the Ebola outbreak in the Democratic Republic of the Congo reflect a lack of sustained international commitment rather than the absence of an intermediate alert. The same primary obstacle applies to the idea of a ‘regional PHEIC’ alert. This idea overlooks the fact that WHO regions are not structured for event-specific management. Such an alert system could result in a fragmented, ‘balkanized’ emergency response system [19], which would fundamentally undermine States Parties’ obligations to support one another regardless of regional affiliation.

### A tier of pandemic emergency: its rationale

The WHO employs its ‘pandemic emergency’ alert framework to synchronize cross-border responses to infectious disease emergencies. On 11 March 2020, the DG declared COVID-19 a ‘pandemic’. Notably, the authority to declare a ‘pandemic’ does not stem from the IHR, but from the 2017 WHO Pandemic Influenza Risk Management Guide, outlining a four-phase global alert system—inter-pandemic, alert, pandemic, and transition phases—evolving from the six-phase alert system introduced in 2009. The 2020 declaration highlighted ambiguity surrounding the term ‘pandemic’, prompting calls for clearer definitions [20].

Unlike the contentious intermediate alert proposal, this higher-tier design gained broad consensus, reflecting agreement on the need for clear jurisdictional guidance in addressing large-scale, cross-border health threats. States Parties advocated for criteria for determining a ‘pandemic’ or ‘pandemic emergency’, emphasizing that such a definition should avoid restrictive criteria that might delay effective public health responses [21]. Ultimately, the amended IHR introduced a ‘pandemic emergency’ tier, specifically defined as a communicable disease in Article 1.

Structurally, this tier extends rather than overhauls the existing PHEIC framework. Articles 12 and 49 of the amended IHR stipulate that a pandemic emergency must first meet the criteria for a PHEIC, aligning its duration with the underlying PHEIC. This design seamlessly integrates the ‘pandemic emergency’ tier into the PHEIC framework, eliminating unnecessary delays and inefficiencies in reconvening the Emergency Committee for the same event, while preserving the dynamic responsiveness intrinsic to the IHR. By embedding a ‘pandemic emergency’ tier, the amended PHEIC framework ensures legal coherence and operational efficiency, representing a significant advancement toward the legal normalization of the WHO’s emergency powers without bureaucratic layers.

## Pandemic emergency: worthy of attention

During emergencies, the WHO’s efforts are most intensely directed toward securing political commitment from Member States, even as the legal certainty of its actions remains comparatively limited. This tension is exemplified by the WHO’s exercise of emergency powers, particularly in its power to determine a PHEIC, including a pandemic emergency.

### A ratchet effect after COVID-19

International organizations (IOs) adopt emergency powers typically resulting in two possible outcomes through rhetorical legitimacy struggles. One is the ratchet effect, where exceptional powers become normalized, either factually or legally, within the IO’s governance structures. The other is the rollback effect, characterized by strong backlash that contains expanded authorities [22]. The proportionality theory provides an analytical framework for evaluating whether an IO’s emergency powers become normalized or contained [23]. The post-SARS amendments to the IHR present the ratchet effect by legally normalizing WHO’s exceptional competencies. Conversely, the post-H1N1 reforms, which decoupled national actions from global influenza phases [24], reflect the rollback effect due to strong backlash against the WHO's expanded authority.

The WHO’s emergency power to determine a pandemic emergency arguably exemplifies a ratchet effect, as evidenced by its formal incorporation into the amended IHR. The declaration of COVID-19 as a pandemic was justified by its global threat and has been proven both necessary and effective. The COVID-19 pandemic underscored the devastating global impact of infectious diseases, reinforcing commitments to develop a new instrument for pandemic prevention, preparedness and response as part of the global health architecture [25]. Moreover, the DG’s determination of COVID-19 as a pandemic effectively raised global alarm. Although WHO leadership initially downplayed the practical implications of labeling COVID-19 a pandemic, the 2021 Review Committee for COVID-19 observed that most countries substantially increased response measures, including coordinating vaccine development and distribution, only after the pandemic declaration. Notably, while the WHO’s emergency power to determine a pandemic expanded in both the H1N1 and COVID-19 cases, outcomes diverged: the former demonstrated limited efficacy, while the latter proved essential and functional.

### Emergency powers in disguise

Alongside the legal normalization of the DG’s emergency power to determine a ‘pandemic emergency’, the amended IHR carries broader implications. The obligations imposed on the WHO by new provisions may, in practice, expand the WHO’s emergency powers following the determination of a PHEIC, including a pandemic emergency.

For example, Article 13(8) introduces obligations requiring the WHO to remove barriers to timely and equitable access to relevant health products after the determination of and during a PHEIC, including a pandemic emergency. Under this provision, the DG is required to:

- conduct periodic assessments of public health needs and issue, modify, extend or terminate such recommendations;
- utilize WHO-coordinated mechanisms;
- upon request, scale up and geographically diversify production;
- upon request, share product dossiers related to specific relevant health products;
- upon request, promote research and development and strengthen local production.

Such obligations do not limit the WHO’s emergency powers; rather, they expand them within the framework of public health response. For instance, the DG’s mandate to conduct periodic assessments of public health needs implicitly authorizes oversight and evaluation of global production and distribution of relevant health products. Moreover, Article 13(9) obliges States Parties to support the WHO in implementing these actions, which further expand the WHO’s access to information regarding relevant health products provided by States Parties.

### A tier of pandemic emergency as a trigger

Introducing a ‘pandemic emergency’ tier within the PHEIC framework serves not only as a normative trigger for activating key mechanisms under the Pandemic Agreement, but also as a litmus test of trust in WHO governance. Once activated, this added tier would invoke key pillars of the Pandemic Agreement, including research and development (Article 9), transfer of technology and cooperation on related know-how for the production of pandemic-related health products (Article 11), pathogen access and benefit-sharing system (Article 12), and procurement and distribution (Article 14). Once the Pandemic Agreement enters into effect, the WHO will likely receive substantial resources, reinforcing its central role in pandemic prevention, preparedness, and response. Consequently, the WHO’s emergency powers must be exercised with heightened accountability, transparency, and effectiveness to sustain and strengthen global trust.

The recent adoption of the Pandemic Agreement by the 78th WHA underscores the imperative for reinforced trust in the WHO’s emergency powers. Emergencies compel actions into two operational spaces: routine space and decision space. For emergencies within the routine space, emergency powers operate under formalized legal frameworks. In contrast, for emergencies within the decision space, emergency powers rely on social trust cultivated during peacetime to legitimize decisions [26]. Article 12 of the IHR provides established criteria for determining emergencies, yet when scientific uncertainty prevails, emergency decision-making enters the decision space. Decisions under uncertainty inherently involve three key paradoxes:

1. The paradox between consequence-oriented emergency decision-making and the high uncertainty of consequences.

2. The paradox between emergency decision-making and the rule of law.

3. The paradox between hindsight science and foresight emergency decision-making [27].

The IHR attempts to bridge these divides through Article 12, which identifies scientific principles, available scientific evidence, and other relevant information, as criteria for the DG’s determination of a PHEIC, including a pandemic emergency. Yet this provision itself reveals a challenge: scientific evidence may often be incomplete or uncertain. Decisions informed by such evidence are inherently provisional, dynamic, and potentially fallible, creating tension between the stability of the rule of law and effective decision-making in emergencies. As a result, decisions made under uncertainty are inherently controversial. Trust in emergency decision-making should therefore be explicitly integrated into the IHR, acknowledging the delegation of emergency powers to the DG as an extension of trust established during peacetime. The WHO’s current reform process represents a complex institutional transformation that cannot be achieved in the short term. Restoring trust among Member States will necessarily require sustained efforts over time, and the institutionalization of the WHO’s emergency powers under the amended IHR represents a crucial step in this gradual process of reform. The amended IHR provides an opportunity for the WHO to demonstrate scientific rigor, transparency, and accountability consistently when exercising emergency powers amidst scientific uncertainty.

## Conclusions

The adoption of the amended IHR marks a watershed moment in global health governance, shaped by the failures of fragmented multilateral responses to COVID-19. These amendments offer a post-COVID-19 reset, shifting global health governance from rhetoric to actionable solidarity. The effective implementation of the WHO’s emergency powers now depends on the collective commitment of Member States, making it imperative to overcome political gridlock and foster more robust and cohesive international collaboration.

## Data Availability

Not applicable.

## Abbreviations

DG: Director-General
IHR: International Health Regulations (2005)
INB: Intergovernmental negotiating body
IO: International Organizations
Pandemic Agreement: WHO convention, agreement, or other international instrument on pandemic prevention, preparedness and response
PHEIC: Public Health Emergency of International Concern
SARS: Severe acute respiratory syndrome
The 2016 Review Committee for Ebola: Review Committee on Role of the IHR in the Ebola Outbreak and Response
The 2017 Pandemic Influenza Guide: The 2017 WHO pandemic influenza risk management guide
The 2021 Review Committee for COVID-19: Review Committee on the Functioning of the IHR during the COVID-19 Response
The 2023 Review Committee for Amendments: Review Committee regarding amendments to the IHR
WGIHR: Working Group on Amendments to the International Health Regulations (2005)
WHA: World Health Assembly
WHO: World Health Organization
